# Impact of Biochar Addition on Biofloc Nitrifying Bacteria and Inorganic Nitrogen Dynamics in an Intensive Aquaculture System of Shrimp

**DOI:** 10.3390/microorganisms12122581

**Published:** 2024-12-13

**Authors:** Wujie Xu, Demin Zhang, Haochang Su, Yu Xu, Xiaojuan Hu, Guoliang Wen, Yucheng Cao

**Affiliations:** 1 South China Sea Fisheries Research Institute, Chinese Academy of Fishery Sciences, Guangzhou 510300, China; su.haochang@163.com (H.S.); xuyublq@163.com (Y.X.); xinr129@163.com (X.H.); wgl610406@163.com (G.W.); cyc_169@163.com (Y.C.); 2 Southern Marine Science and Engineering Guangdong Laboratory (Zhuhai), Zhuhai 519082, China; 3 State Key Laboratory for Managing Biotic and Chemical Threats to the Quality and Safety of Agro-Products, Ningbo University, Ningbo 315000, China

**Keywords:** shrimp, biochar, biofloc, bacterial community, nitrifying bacteria, nitrifying gene, nitrogen transformation, nitrogen dynamic

## Abstract

In this study, an eight-week culture trial of *Penaeus vannamei* juveniles was conducted in commercial intensive systems to compare the impacts of biochar and molasses addition on biofloc nitrifying bacteria and inorganic nitrogen dynamics under limited water exchange conditions. During the trial, the biofloc concentration (in terms of VSS and TSS), quantities of total bacteria (TB) and total *Vibrio* (TV), and ratio of TV/TB in the culture water were lower in the biochar group compared to the molasses group. Metagenomic sequencing analysis revealed that the bacterial community composition of bioflocs showed higher α-diversity and complexity in the biochar group compared to the molasses group. Moreover, the abundance of nitrifying bacterial genera and functional genes in bioflocs was higher in the biochar group compared to the molasses group. Inorganic nitrogen dynamics showed that NH_4_^+^-N and NO_2_^−^-N were better controlled in the biochar group compared to the molasses group, as reflected by lower peaks of NH_4_^+^-N and NO_2_^−^-N and higher NO_3_^−^-N concentrations. Excellent production performance of shrimp was achieved, which in turn proved the reliable effect of biochar addition on the mediation of inorganic nitrogen transformation through nitrifying bacteria. These results showed that biochar addition could promote biofloc nitrifying bacteria and nitrification to more effectively control harmful nitrogen for shrimp efficient production. This study provides a practical example for the biochar application in biofloc-based systems for intensive aquaculture.

## 1. Introduction

Shrimp aquaculture has become a leading seafood production industry worldwide, and is one of the main aquaculture activities in many countries and coastal regions [1]. Facing a huge demand of consumption and shortage of water resources, shrimp aquaculture has developed rapidly with increasing intensification and large-scale operations in recent decades, especially in Asian countries, such as China, Vietnam, India, and Indonesia [1]. Intensive aquaculture of shrimp relies heavily on high-protein feed input into the culture systems. As the utilization of feed protein by shrimp is limited, the water environment of intensive systems can rapidly deteriorate due to endogenous nitrogenous wastes during the production process [2,3]. In particular, harmful nitrogenous substances, such as ammonia and nitrite, can usually accumulate in the culture water, which poses threats to shrimp survival and growth [4]. In practice, water is exchanged at a high frequency and in large quantities to maintain the water quality of the aquaculture systems [5]. This operation not only wastes water resources but also pollutes the surrounding waters [6,7]. Maintaining water quality in intensive systems with limited or zero-water exchange is still an urgent problem that needs to be solved for the sustainable development of shrimp aquaculture [8,9,10].

In recent years, biofloc-based systems have gained significant attention for their strong performance on water quality control in shrimp-intensive aquaculture [11,12,13]. Bioflocs can naturally form from various active bacteria with accumulated organic particles, and then suspend in the culture water to act as the functional core of system operation [14]. Previous studies have shown that bioflocs can transform ammonium–nitrogen (NH_4_^+^-N) for the maintenance of water quality through microbial processes such as heterotrophic bacterial assimilation and autotrophic bacterial nitrification [15,16]. Under limited or zero-water exchange, bioflocs can continually produce and accumulate in the culture water of intensive systems, which may then bring problems such as increased oxygen consumption, sharply reduced pH, and fluctuating NH_4_^+^-N and nitrite–nitrogen (NO_2_^−^-N) concentrations [17,18]. Therefore, determining an appropriate range of biofloc concentration in the culture water is necessary for the smooth and sufficient operation performance of biofloc-based systems [16,18,19].

Organic carbon addition is a common operation means for the development and management of bioflocs in intensive aquaculture systems [20]. Molasses is usually used to stimulate and accelerate the formation of bioflocs in shrimp aquaculture systems [21,22]. In practice, a large amount of molasses needs to be added into the culture water for heterotrophic bacteria growth and biofloc promotion [16,17,19]. Biochar, as a bio-carbon-rich material widely used in environmental applications, can be used as a new kind of organic carbon source in aquaculture [23,24]. Biochar can be produced by thermal decomposition of agricultural wastes under oxygen-limited conditions, and has unique features such as high carbon content, slow-release property, large surface area, and stable porous structure [23]. These properties indicate that biochar may be an excellent organic carbon material for application in biofloc aquaculture systems [24]. Moreover, previous studies have shown that biochar can stimulate the growth of nitrifying bacteria, thereby positively influencing nitrification performance in environmental systems [25].

It is widely acknowledged that the bacterial communities within bioflocs play important roles in N-transformation and nitrogen dynamics in intensive aquaculture systems [14,16]. Specifically, the composition and abundance of nitrifying bacteria in bioflocs should be promoted to effectively control NH_4_^+^-N and NO_2_^−^-N in the culture water for shrimp aquaculture [16,19,26]. In order to explore the application of biochar in shrimp production systems, an eight-week culture trial of *Penaeus vannamei* was conducted in commercial intensive systems with molasses and biochar addition under limited water exchange conditions. The following aspects were investigated and compared: (1) the change in biofloc concentration and *Vibrio* quantity in the culture water; (2) the composition and diversity of bacterial communities in the bioflocs; (3) the composition and abundance of nitrifying bacteria and genes in the bioflocs; (4) the dynamics of inorganic nitrogen and production performance of shrimp in the biofloc systems. The results of this study will provide theoretical and technical foundations for the application of biochar in biofloc-based systems for shrimp-intensive aquaculture.

## 2. Materials and Methods

### 2.1. Biofloc-Based System and Shrimp Stocking

The study was conducted in biofloc-based systems, which were constructed in a greenhouse with semi-shaded plastic film. Each system consisted of a concrete tank (water volume of 30 m^3^), nine water injectors, a 750 W circulating pump, and a small-size foam fractionator (Figure S1). The water source was natural seawater, which was pumped into each culture tank after being sand-filtered and chlorinated. Before the trial, the mature biofloc-rich water from the previous batch was pumped and inoculated into each system at a volume ratio of 5%. Twelve biofloc-based systems were prepared, each stocked with juvenile shrimp *P. vannamei* (2.33 ± 0.11 g) at a density of 520 shrimp m^−3^. The trial began and lasted for eight weeks.

### 2.2. Trial Design and Culture Management

The trial was divided into two groups based on the type of organic carbon source added. Agricultural biochar and molasses were used, and their basic characteristics are presented in Table S1. The biochar was purchased from Zhejiang Ai Green Chemical Technology Co., Ltd. (Quzhou, Zhejiang, China), and was prepared by pyrolyzing at 500 °C, and then pulverizing and sieving using corn stalk as raw material. The molasses, containing more than 95% sucrose, was purchased from a sugarcane plant. Each of the two carbon sources was applied in six randomly assigned biofloc-based systems. For every 1.0 kg of the feed offered in the biofloc system during the trial, 0.24 kg of biochar and 0.40 kg of molasses were added into the system, respectively, to achieve a carbon/nitrogen (C/N) ratio of 10 based on the carbon and nitrogen contents of both the input feed and carbon source [17]. The addition of biochar or molasses was stopped as both NH_4_^+^-N and NO_2_^−^-N in the tank water were detected to be below 1.0 mg L^−1^ during the trial.

The shrimp feed was purchased from Guangdong Guangxin Feed Co., Ltd. (Maoming, Guangdong, China), which had 40.1% protein, 8.4% lipid, 3.5% fiber, and 13.5% ash. The feeds were offered by automatic feeders twelve times daily, and the daily rations decreased from 12% to 3% during the trial based on the estimation of shrimp biomass and the observation of feeding trays [19]. During the trial, no water of the system was exchanged in the first three weeks, and thereafter, 2~5% of culture water was exchanged daily to maintain biofloc levels within appropriate ranges. Agricultural sodium carbonate (purity > 95.0%) was added into the tank water to maintain a pH above 7.0 as needed during the trial. The amount of offered feed, exchanged water, used carbon sources, and added sodium carbonate were recorded during the trial.

### 2.3. Water Quality Monitoring and Biofloc Quantitative Evaluation

The daily monitoring parameters included salinity, temperature, dissolved oxygen, and pH. The results are presented in Table S2. Every week during the trial, water samples were collected from each system to analyze total suspended solids (TSS), volatile suspended solids (VSS), NH_4_^+^-N, NO_2_^−^-N, and nitrate–nitrogen (NO_3_^−^-N) following the “Standard methods for the examination of water and wastewater” [27]. Here, NH_4_^+^-N represents total ammonium–nitrogen, including ionized (NH_4_^+^) and unionized (NH_3_) forms of ammonium–nitrogen in the water sample. The TSS and VSS were used to evaluate biofloc concentrations in the culture water [17].

### 2.4. Quantification of Total Bacteria and Total Vibrio in Culture Water

Every week during the trial, total bacteria (TB) and total *Vibrio* (TV) were determined using real-time fluorescence quantitative PCR detection kit, following the instructions (Xiamen Zhihui Lianfeng Biotechnology Co., Ltd., Xiamen, China) [28]. The bacterial 16S rRNA and *rpoD* gene were targeted for the detection of total bacteria and total *Vibrio* [29]. Briefly, 15 mL of water sample was taken from the culture tank of each system and then filtered through a polycarbonate membrane filter with a pore size of 0.22 μm (Merck Millipore, Burlington, MA, USA). The genomic DNA from the filters was extracted and then subjected to real-time fluorescence quantification PCR assay. The PCR reaction was carried out using 20 μL reaction mixture that contained 10 μL of 2× qPCR mix, 8 μL of fluorescence probe, and 2 μL of template DNA. The cycling parameters consisted of an initial denaturation at 95 °C for 2 min, followed by 40 cycles of amplification, with each cycle consisting of denaturation at 94 °C for 10 s and a combined annealing/extension step at 60 °C for 30 s. The quantities of total bacteria and total *Vibrio* in culture water were normalized gene copy numbers to per milliliter water volume.

### 2.5. Biofloc DNA Extraction, Metagenomic Sequencing, and Bioinformatics Analysis

At the end of the trial, 50 mL of culture water was collected from each system and then filtered through a polycarbonate membrane filter with a pore size of 0.45 μm (Merck Millipore, USA) to obtain a biofloc sample. Total genomic DNA was extracted from biofloc samples using the E.Z.N.A.^®^ Soil DNA Kit (Omega Bio-tek, Winooski, VT, USA); and after the measurement of quantity and quality, the extracted DNA was then sent for metagenomics sequencing at Wekemo Tech Group Co., Ltd., Shenzhen, China. Individual libraries were constructed using the NEBNext@ UltraTM DNA Library Prep Kit (NEB, Ipswich, MA, USA), and DNA sequencing was performed on the Illumina NovaSeq 6000 platform (Illumina, San Diego, CA, USA) using a 2 × 150 bp paired-end read protocol. The raw sequence data generated in this study have been deposited into the NCBI Short Read Archive database (accession number: PRJNA1187254).

The raw sequences were preprocessed using Kneaddata (v0.7.4 https://github.com/biobakery/kneaddata, accessed on 18 November 2024) for quality control. Then, all clean sequences were annotated and classified using kraken2 (v2.0.8-beta, http://ccb.jhu.edu/software/kraken2/, accessed on 18 November 2024) and a self-built microbial database (sequences were screened from the NT nucleic acid database and RefSeq whole genome database of NCBI) to characterize the taxonomic composition of the metagenomic dataset of the samples. Bracken (v2.0, https://ccb.jhu.edu/software/bracken/index.shtml, accessed on 18 November 2024) was used to estimate the species-level abundance of metagenomic samples.

The clean sequences were also assembled into contigs using Megahit (v1.2.9, http://www.l3-bioinfo.com/products/megahit.html, accessed on 18 November 2024), and gene sequences in all contigs were predicted using METAProdigal (v2.6.3, https://github.com/hyattpd/prodigal, accessed on 18 November 2024). Then, the de-redundant gene was obtained using Cd-hit (v4.8.1, http://www.bioinformatics.org/cd-hit/, accessed on 18 November 2024), quantified using Salmon (v0.13.1, https://github.com/COMBINE-lab/salmon, accessed on 18 November 2024), and translated into protein sequences for subsequent blast and functional annotation against the Kyoto Encyclopedia of Genes and Genomes (KEGG v94.2, http://www.genome.jp/kegg/, accessed on 18 November 2024). The targeted N-cycling genes were filtered out from the metagenomic samples. The abundance of the de-redundant gene was annotated to the same gene family and presented as Transcripts Per Kilobase Million (TPM) [30].

### 2.6. Shrimp Harvest and Performance Determination

At the end of the trial, the water of each tank was drained. Shrimp were harvested with dip nets into baskets, and then weighed to obtain the total harvest biomass. Meanwhile, two hundred shrimps from each tank were randomly chosen for the determination of harvest weight. The indicators of production performance were harvest weight (g), growth rate (g week^−1^), survival rate (%), yield (kg m^−3^ water), feed conversion ratio, carbon source usage (kg kg^−1^ shrimp), sodium carbonate usage (kg kg^−1^ shrimp), and water usage (L kg^−1^ shrimp), which were calculated following the methods of our previous study [19].

### 2.7. Statistical Analysis

All statistical analyses were performed using IBM SPSS Statistics 20.0 software for Windows (IBM Corporation, Armonk, NY, USA). The data of the parameters of biofloc concentration (VSS and TSS), bacterial quantity (TB, TV, and ratio of TV/TB), and N dynamics (NH_4_^+^-N, NO_2_^−^-N, and NO_3_^−^-N) were analyzed by Linear Mixed Models, using Factor Analysis (First-Order, Heterogeneous) with treatment and time as fixed effects [17]. Furthermore, for these parameters, comparisons between the two treatments were made at each sampling time using Student’s *t*-test. The data of other parameters were compared between the two treatments using Student’s *t*-test. All data were checked for normality using the Shapiro–Wilk test, and homogeneity of variance was examined using Levene’s test. The statistical difference was considered significant if *p* < 0.05. The percentage data were transformed with arcsine square root before analysis.

## 3. Results

### 3.1. Biofloc Concentration and Bacterial Quantity Changes in Shrimp Culture Systems

**Figure 1:**
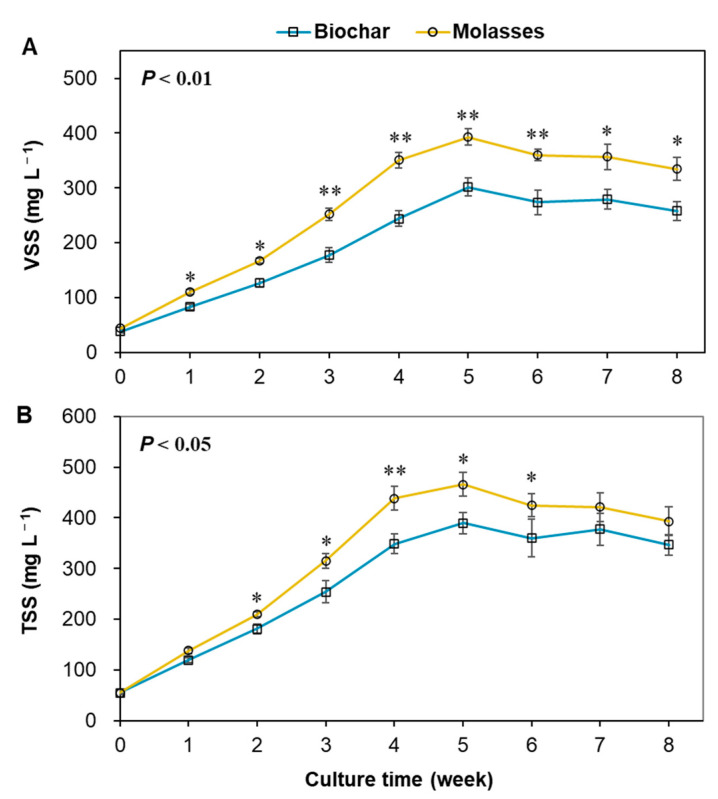
Change in biofloc concentration in the culture water of biofloc systems with the addition of biochar and molasses in an 8-week culture trial of shrimp (means ± S.D., *n* = 6). (**A**): VSS—volatile suspended solids; (**B**): TSS—total suspended solids. The asterisk (*) indicates a significant difference between the two groups (* *p* < 0.05, ** *p* < 0.01).

During the trial, the biofloc concentrations in terms of VSS and TSS increased rapidly before week 4 and thereafter were maintained below 500 mg L^−1^ and 400 mg L^−1^, respectively, in the two groups (Figure 1). Both VSS and TSS showed significantly lower levels in the culture water with biochar compared to that with molasses (*p* < 0.05).

**Figure 2:**
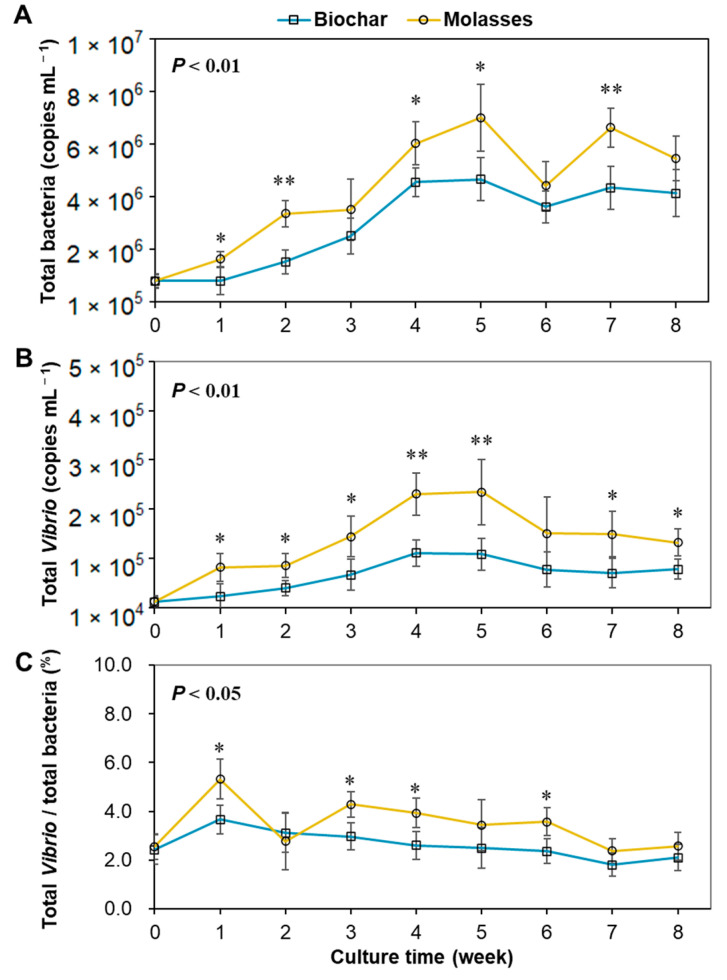
Quantity changes in total bacteria and total *Vibrio* in the culture water of biofloc systems with the addition of biochar and molasses in an 8-week culture trial of shrimp (means ± S.D., *n* = 6). (**A**): Total bacteria; (**B**): total *Vibrio*; (**C**): ratio of total *Vibrio*/total bacteria. The asterisk (*) indicates a significant difference between the two groups (* *p* < 0.05, ** *p* < 0.01).

Along with the development of biofloc in the culture water, the quantities of TB and TV were both increased gradually before week 4 and thereafter remained around 5 × 10^6^ copies mL^−1^ and 1 × 10^5^ copies mL^−1^, respectively, in the two groups (Figure 2). Both TB and TV showed significantly lower quantities in the culture water with biochar than that with molasses (*p* < 0.01). Meanwhile, the ratios of TV/TB were between 1.8% and 5.3% in the two groups, which were significantly lower in the biochar group compared to the molasses group (*p* < 0.05).

### 3.2. Inorganic Nitrogen Dynamics and Shrimp Production Performance in Culture Systems

**Figure 3:**
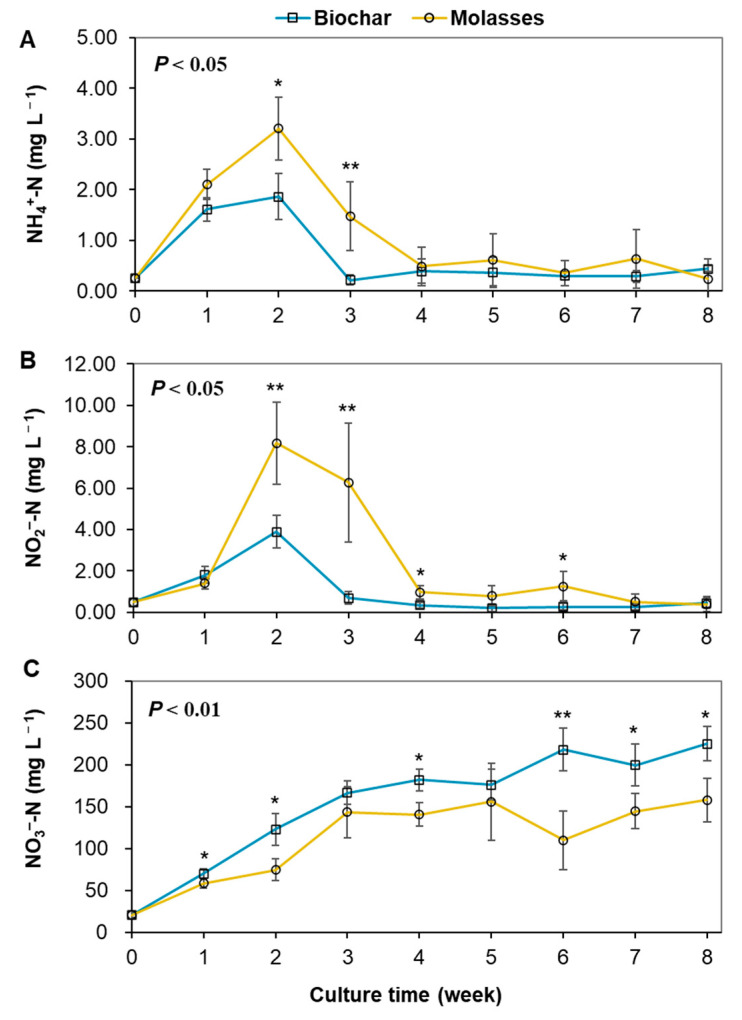
Inorganic nitrogen dynamics in the culture water of biofloc systems with the addition of biochar and molasses in an 8-week culture trial of shrimp (means ± S.D., *n* = 6). (**A**): NH_4_^+^-N; (**B**): NO_2_^−^-N; (**C**): NO_3_^−^-N. The asterisk (*) indicates a significant difference between the two groups (* *p* < 0.05, ** *p* < 0.01).

During the trial, both TAN and NO_2_^−^-N concentrations increased rapidly in the first two weeks, and then decreased rapidly and remained below 1.0 mg L^−1^ in the two groups (Figure 3). Both NH_4_^+^-N and NO_2_^−^-N concentrations peaked at around week 2, reaching 1.86 mg L^−1^ and 3.21 mg L^−1^ of NH_4_^+^-N and 3.89 mg L^−1^ and 8.18 mg L^−1^ of NO_2_^−^-N in the biochar and molasses groups, respectively. As a whole, both NH_4_^+^-N and NO_2_^−^-N concentrations showed significantly lower (*p* < 0.05) levels in the culture water in the biochar group compared to the molasses group, especially for the peaks. The NO_3_^−^-N concentrations showed gradual and fluctuating increases in the two groups, with significantly higher levels in the culture water with biochar than that with molasses (*p* < 0.01).

After the trial, shrimp harvest weight, growth rate, survival rate, yield, and feed conversion ratio showed no significant difference between the two groups (*p* > 0.05) (Table 1). Significant lower usage amounts of carbon source, sodium carbonate, and water for producing 1 kg of shrimp were found in the biochar group compared to the molasses group (*p* < 0.05) (Table 1).

### 3.3. Bacterial Communities and N-Transformation Pathways of Bioflocs in Culture Systems

**Figure 4:**
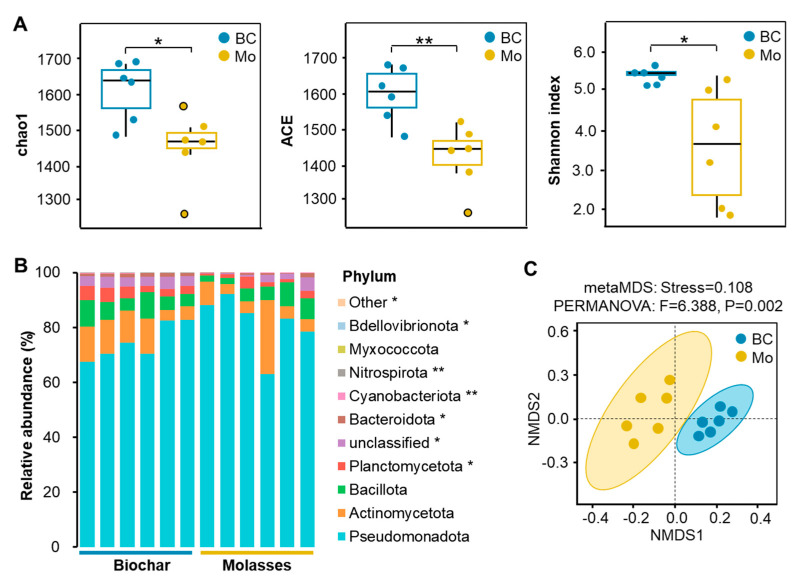
Diversity and composition of bacterial communities in the bioflocs with the addition of biochar and molasses in an 8-week culture trial of shrimp (means ± S.D., *n* = 6). (**A**): Bacterial alpha diversity; (**B**): bacterial composition at phylum level; (**C**): bacterial beta diversity. The asterisk (*) indicates a significant difference between the two groups (* *p* < 0.05, ** *p* < 0.01).

Significant differences were found in the diversity and composition of bacterial communities in the bioflocs between the biochar and molasses groups (Figure 4). The addition of biochar significantly increased species richness (Chao1 and ACE) and alpha diversity (Shannon index) of the bioflocs compared to molasses (*p* < 0.05) (Figure 4A). Among the top ten bacterial phyla, Planctomycetota, Bacteroidota, Cyanobacteriota, Nitrospirota, and Bdellovibrionota had significantly higher relative abundances in the biochar group compared to the molasses group (*p* < 0.05) (Figure 4B). Pseudomonadota was by far the dominant bacterial phylum in the bioflocs, accounting for 74.7% and 81.7% of the entire community composition in the biochar and molasses groups, respectively. Beta diversity analysis further showed there was highly significant difference in the distribution of bacterial communities between the biochar and molasses groups (*p* < 0.01) (Figure 4C).

**Figure 5:**
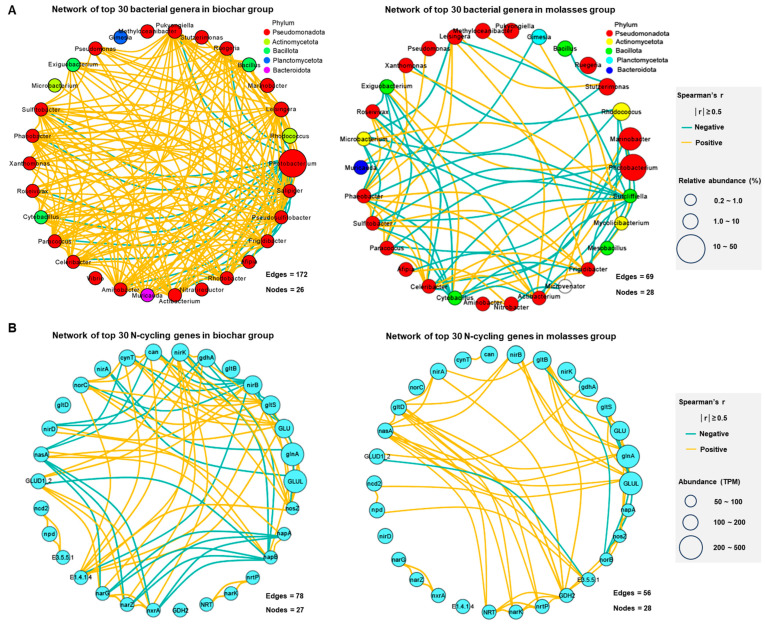
Correlation networks of dominant bacterial genera and N-cycling genes in the bioflocs with the addition of biochar and molasses in an 8-week culture trial of shrimp (means ± S.D., *n* = 6). (**A**): Network of the top 30 bacterial genera in the biochar and molasses groups; (**B**): network of top 30 N-cycling genes in the biochar and molasses groups.

There were significant differences in the correlation networks of dominant bacterial genera and N-cycling genes in the bioflocs between the biochar and molasses groups (Figure 5). The addition of biochar significantly increased the connections among the top 30 bacterial genera of the bioflocs compared to molasses, and most of them were significantly positive relations (Figure 5A). The genera of *Photobacterium*, *Pseudosulfitobacter*, and *Leisingera* had the highest number of connections in the biochar group while the genera of *Sutcliffiella*, *Cytobacillus*, and *Frigidibacter* had the highest number of connections in the molasses group. Meanwhile, the addition of biochar significantly increased the connections among the top 30 N-cycling genes of the bioflocs compared to molasses (Figure 5B). The genes of *napB* and *nirB* had the highest number of connections in the biochar group while the genes of *GDH2* and *GLUL* had highest number of connections in the molasses group.

**Figure 6:**
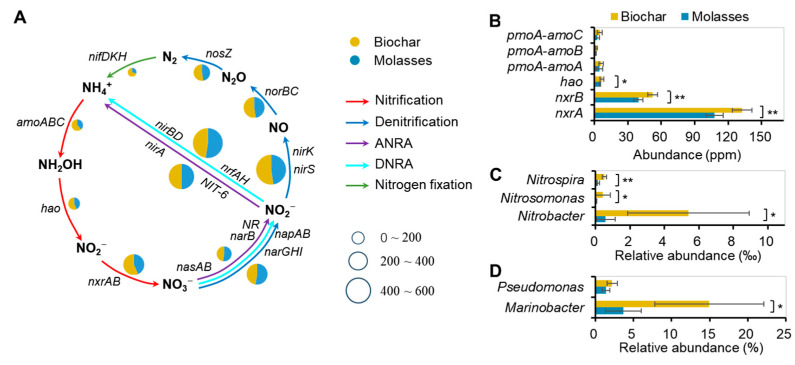
Inorganic N-transformation pathways and involved functional genes and bacterial genera in the bioflocs with the addition of biochar and molasses in an 8-week culture trial of shrimp (means ± S.D., *n* = 6). (**A**): Inorganic N-transformation pathways and genes; (**B**): nitrifying functional genes; (**C**): autotrophic nitrifying bacterial genera; (**D**): heterotrophic nitrifying bacterial genera. The asterisk (*) indicates a significant difference between the two groups (* *p* < 0.05, ** *p* < 0.01).

A complete inorganic N-cycling network was constructed based on functional genes identified from the biofloc (Figure 6, Table S3), and it comprised five N-transformation pathways: nitrification, denitrification, assimilatory nitrate reduction to ammonium (ANRA), dissimilatory nitrate reduction to ammonium (DNRA), and nitrogen fixation (Figure 6A). The addition of biochar could significantly enhance the nitrification process, as indicated by the higher abundances of nitrifying genes (*hao*, *nxrA*, and *nxrB*) compared to molasses (Figure 6B). Moreover, the biochar group had significantly higher relative abundances in the autotrophic nitrifying bacterial genera (*Nitrosomonas*, *Nitrobacter*, *and Nitrospira*) and heterotrophic nitrifying bacterial genera (*Marinobacter* and *Pseudomonas*) compared to the molasses group (Figure 6C,D).

## 4. Discussion

### 4.1. Biochar Controls Biofloc Concentration and Vibrio Quantity in Shrimp Culture Systems

Biofloc concentration is usually quantified by TSS and VSS, which have important effects on shrimp performance, water quality, and microbial community [16,17,18]. In this study, the bioflocs were well-developed and well-managed, as reflected by the gradual increases in VSS and TSS levels during the early stages, followed by their maintenance at moderate levels through limited water exchange in the later stages of the trial. The addition amount of biochar was significantly less than molasses in the trial, which provided less organic carbon for heterotrophic bacteria growth and biofloc production, thereby resulting in a lower biofloc concentration in the biochar group compared to the molasses group [17,26]. Meanwhile, compared to the molasses group, a lower amount of sodium carbonate was added into the culture water for pH adjustment, and a lower amount of water was exchanged to remove and control the bioflocs for shrimp culture in the biochar group during the trial. Therefore, the addition of biochar could be beneficial for biofloc development and concentration control in limited water exchange systems for shrimp-intensive culture.

*Vibrio* spp. are potential opportunistic pathogens in aquaculture systems [31]; therefore, it is necessary to monitor *Vibrio* quantity in the biofloc systems from the standpoint of disease prevention and control [32]. In this study, the TV quantity and the ratio of TV/TB were well controlled in the biofloc systems, and the addition of biochar led to better performance compared to molasses. This suggests the advantage of biochar used as a carbon source in biofloc systems. During the trial, the lower addition amount of biochar and its slow-release properties brought lower biofloc concentration and bacterial density, which provided limited nutritional environments for the proliferation of r-strategist bacteria (e.g., *Vibrio* spp.) [33]. Moreover, the ratios of TV/TB were maintained below 5% in the two groups during the trial, which are generally considered safe levels in the practice of shrimp aquaculture [34]. The addition of biochar showed a better control effect on *Vibrio* quantity and the ratio of TV/TB, necessitating further studies to explore the underlying mechanism in biofloc systems.

### 4.2. Biochar Improves Bacterial Community Diversity of Biofloc in Shrimp Culture Systems

Previous studies have demonstrated that different carbon sources have effects on the bacterial community diversity and composition in biofloc systems [20]. In this study, higher species richness and alpha diversity of bacterial community were observed in the bioflocs with biochar addition compared to molasses. It is deduced that the availability and complexity of carbon sources could affect the bacterial community diversity of bioflocs [35,36]. The higher addition amount of molasses and its higher availability of organic carbon could be beneficial for fast-growing species (r-strategists) and thereby inhibit species richness and community diversity, while the lower addition amount of biochar and its lower availability of organic carbon could be beneficial for slow-growing bacteria (K-strategists) and thereby strengthen species richness and community diversity [18,37]. This could also be the reason for the differences in the abundance and distribution of bacterial communities in the bioflocs between the two groups as observed in the trial. Furthermore, higher abundances of Planctomycetota, Bacteroidota, Cyanobacteriota, and Nitrospirota in the bioflocs potentially contributed to the enhancement of nitrification, denitrification, DNRA, and nitrogen fixation in the biochar group compared to the molasses group [38,39]. Thus, the increased bacterial diversity in biofloc systems with biochar addition could have led to more efficient nitrogen transformation and improved ecosystem stability [14].

The addition of biochar also increased the connection and complexity of bacterial communities and N-cycling genes in the bioflocs in this study. There were more connections, and most of them were positive relations among bacterial genera in the bioflocs with biochar addition compared to molasses, which suggests that more cooperative interactions existed and helped to form a stable ecological network [40]. This, in turn, corroborated the higher diversity of bacterial communities observed in the bioflocs with biochar addition. Meanwhile, the bacterial network core genera (the nodes of highest connection number) changed and could play important roles in maintaining the stability of bacterial communities in the biofloc systems. The genera of *Photobacterium*, *Pseudosulfitobacter*, and *Leisingera* are commonly found to be involved in N-transformation in the biofloc systems [16]. There were more connections of N-cycling genes in the bioflocs with biochar addition compared to molasses, further suggesting a stable microbial function of N-transformation [16]. A higher level of network connectivity was achieved between bacterial genera and N-cycling genes as a result of the addition of biochar, indicating the enhanced effectiveness of the microorganisms and their functions in biofloc systems, particularly with regard to nitrogen transformation and cycling [16,40]. Moreover, inorganic nitrogen transformation in the biochar group and organic nitrogen transformation in the molasses group should be key pathways of N-recycling in the biofloc-driven N-cycling, as deduced from the N-cycling gene network [16,17].

### 4.3. Biochar Increases Nitrifying Bacteria and Gene of Biofloc in Shrimp Culture Systems

Nitrifying bacteria should play a dominant role in inorganic N-transformation in biofloc systems [15,16,26]. Five N-transformation pathways including nitrification, denitrification, ANRA, DNRA, and nitrogen fixation constitute a complete inorganic N-cycling network in two biofloc systems [41]. This further demonstrated that efficient biofloc systems were established based on the fundamental function of microbial N-cycling [16,42]. Higher abundances of nitrifying genes (*hao*, *nxrA,* and *nxrB*) were found in the biochar group compared to the molasses group, indicating that nitrification process should play a more prominent role in the transformation of NH_4_^+^-N and NO_2_^−^-N to NO_3_^−^-N. This corresponded to higher abundances of Planctomycetota and Nitrospirota in the bioflocs of the biochar group compared to the molasses group, resulting in lower levels of NH_4_^+^-N and NO_2_^−^-N and higher levels of NO_3_^−^-N in the systems. Strong nitrification function of the bioflocs efficiently drove the transformation of NH_4_^⁺^-N and NO_2_^−^-N to NO_3_^−^-N, thereby effectively controlling harmful NH_4_^⁺^-N and NO_2_^−^-N within low levels in the culture water. This is a prerequisite for the smooth and effective operation of biofloc systems for intensive shrimp culture [15,19,26].

Not only autotrophic nitrifying bacteria but also heterotrophic nitrifying bacteria are responsible for the nitrification process in biofloc systems [15,16]. It is interesting to have found that both autotrophic nitrifying bacteria (main genera of *Nitrosomonas, Nitrobacter, and Nitrospira*) and heterotrophic nitrifying bacteria (main genera of *Marinobacter* and *Pseudomonas*) showed higher relative abundances in the biochar group compared to the molasses group. *Nitrosomonas,* a genus of ammonia-oxidizing bacteria (AOB), oxidized NH_4_^+^-N to NO_2_^−^-N, while *Nitrobacter*, a genus of nitrite-oxidizing bacteria (NOB), oxidized NO_2_^−^-N to NO_3_^−^-N; and the combination of them transformed NH_4_^+^-N to NO_3_^−^-N in the culture water of biofloc systems [16,43]. Meanwhile, *Nitrospira* can directly oxidate NH_4_^+^-N to NO_3_^−^-N, also contributing to nitrification process [16,44]. From the perspective of organic carbon supply, a smaller amount and lower availability of organic carbon was introduced by the addition of biochar into the culture water compared to molasses, which provided a water environment with lower organic carbon, favoring the proliferation and growth of autotrophic nitrifying bacteria rather than heterotrophic nitrifying bacteria [45]. Moreover, it is worth noting that the relative abundance of heterotrophic nitrifying bacteria (up to 15%) was far higher than autotrophic nitrifying bacteria (up to 5‰). Anyway, the increased relative abundances of autotrophic and heterotrophic nitrifying bacteria in the bioflocs of biochar group imply that a sufficient nitrogen processing capacity could be achieved and improved in biofloc systems. Further studies are needed to explore and evaluate the contributions of the autotrophic and heterotrophic pathways to the nitrification process in biofloc systems.

### 4.4. Biochar Promotes Nitrification to Control Harmful Nitrogen in Shrimp Culture Systems

Nitrification should be the main pathway for harmful nitrogen (NH_4_^+^-N and NO_2_^−^-N) transformation and control in biofloc systems [15,16,26]. Based on the inorganic nitrogen dynamics, the produced NH_4_^+^-N was continually converted to NO_3_^−^-N through the nitrification process. The bacterial community structure and function analysis further confirmed various nitrifying bacteria and related functional genes existed in the bioflocs, which mediated nitrification in the culture water of biofloc systems. During the trial, harmful nitrogen (NH_4_^+^-N and NO_2_^−^-N) in the culture water was effectively controlled, which guaranteed a good water quality for the survival and healthy culture of shrimp [19,46]. Therefore, excellent production performance of shrimp was achieved in the biofloc systems with the addition of biochar and molasses in this study, compared to those previously published reports [47,48,49]. Based on the addition dosage and control effect, biochar is superior to molasses in promoting nitrification to control harmful nitrogen in the intensive culture of shrimp in biofloc systems.

## 5. Conclusions

The trial conducted in this study demonstrated that biochar addition promoted the nitrification of bioflocs to effectively control harmful nitrogen, thereby sustaining efficient and healthy culture of shrimp in limited water exchange systems. Compared to molasses, a lower amount of biochar addition was sufficient to control biofloc concentration and *Vibrio* quantity in the culture water, improving bacterial community diversity and nitrifying the bacteria abundance of bioflocs. Excellent production performance of shrimp was achieved, which in turn proved the reliable effect of biochar addition for the mediation of inorganic nitrogen transformation through nitrifying bacteria in the trial. Further studies are needed to explore the addition timing and level of biochar for directional construction of nitrifying bioflocs and smooth control of harmful nitrogen in shrimp-intensive aquaculture systems. The application of biochar in this study provides a theoretical basis and practical example in the transformation and control of harmful nitrogen in biofloc systems for intensive aquaculture.

## Figures and Tables

**Table 1:** Production performance of *P. vannamei* in the biofloc systems with the addition of biochar and molasses in an 8-week trial (means ± S.D., *n* = 6).

Indicator	Biochar	Molasses	*p* Value
Harvest weight (g)	17.6 ± 0.4	17.7 ± 0.6	0.68
Growth rate (g week^−1^)	1.90 ± 0.05	1.92 ± 0.08	0.68
Survival rate (%)	87.3 ± 2.6	83.3 ± 4.4	0.09
Yield (kg m^−3^)	7.97 ± 0.29	7.66 ± 0.29	0.08
Feed conversion ratio	1.21 ± 0.04	1.19 ± 0.03	0.45
Carbon source usage ^#^ (kg kg^−1^ shrimp)	0.07 ± 0.01	0.17 ± 0.02	0.00
Sodium carbonate usage (kg kg^−1^ shrimp)	0.04 ± 0.00	0.23 ± 0.01	0.00
Water usage (L kg^−1^ shrimp)	355 ± 18	417 ± 25	0.02

^#^ Carbon source refers to biochar or molasses.

## Data Availability

The raw read datasets for this research can be found in the NCBI Sequence Read Archive database (Accession No. PRJNA1187254) (https://www.ncbi.nlm.nih.gov/sra/ (accessed on 18 November 2024)).

## Supplementary Materials

The following supporting information can be downloaded at: https://www.mdpi.com/article/10.3390/microorganisms12122581/s1, Figure S1: Schematic diagram of the biofloc-based system with shrimp-intensive aquaculture in this study; Table S1: Basic characteristics of biochar and molasses used in this study; Table S2: The daily monitored parameters of the biofloc systems with the addition of biochar and molasses in an 8-week trial of *P. vannamei*; Table S3: N-cycling functional genes and related main bacterial genera identified from the metagenomes of the biofloc in this study.
