# Supplementary material for: Impact of Biochar Addition on Biofloc Nitrifying Bacteria and Inorganic Nitrogen Dynamics in an Intensive Aquaculture System of Shrimp
Source: Microorganisms. 2024 Dec 13;12(12):2581. doi: 10.3390/microorganisms12122581 (PMC11676986; doi:10.3390/microorganisms12122581)
Supplement: Supplementary file 1 [file microorganisms-12-02581-s001.zip › microorganisms-3358156-supplementary.pdf]

Figure S1. Schematic diagram of the biofloc-based system with intensive aquaculture of *Peneaus vannamei* in this study

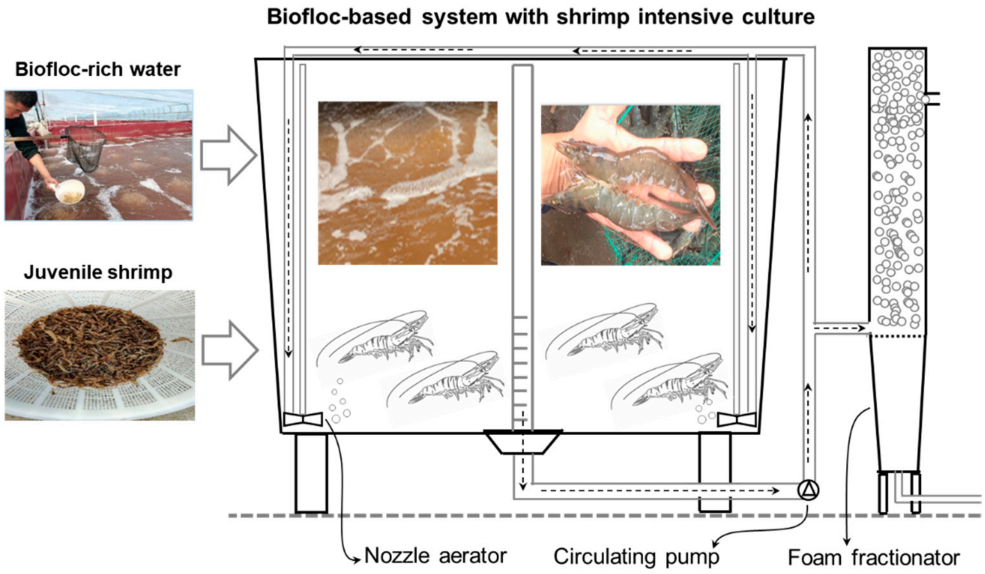

Table S1 Basic characteristics of biochar and molasses used in this study

Item	Biochar	Molasses
Particle size (mm)	0.1~0.5	0.2~1.0
pH	8.2	4.5
Total carbon (%)	67.2	42.0
Total nitrogen (%)	0.78	0.64
Carbon/nitrogen ratio	86.2	65.6

Table S2 The daily monitored parameters of the biofloc systems with the addition of biochar and molasses in an 8-week trial of *P. vannamei* (means ± S.D., n = 6)

Parameter	Biochar		Molasses	
	Mean ± S.D.	Min – Max	Mean (± S.D.)	Min – Max

Light intensity (lx)	3354 ± 56	830 – 6548	3372 ± 68	848 – 6624
Salinity (g L^{-1})	23.9 ± 0.1	22.2 – 26.1	24.1 ± 0.1	22.0 – 26.2
Temperature (°C)	29.3 ± 0.2	26.3 – 32.6	29.4 ± 0.2	26.2 – 32.7
Dissolved oxygen (mg L^{-1})	5.9 ± 0.1	4.6 – 6.9	5.5 ± 0.1	4.4 – 6.8
pH	7.5 ± 0.1	7.3 – 7.9	7.3 ± 0.2	7.1 – 7.9

Table S3 N-cycling functional genes and related main bacterial genera identified from the metagenomes of the biofloc in this study.

Pathway	Process	Gene (sub) family	Annotation	EC code	Dominant genus
Nitrification	$NH_4^+ \rightarrow NH_2OH$	*pmoA-amoA*	methane/ammonia monooxygenase subunit A	EC 1.14.18.3	*Nitrosomonas*
		pmoB-amoB	methane/ammonia monooxygenase subunit B	/	*Nitrosomonas*
		pmoC-amoC	methane/ammonia monooxygenase subunit C	/	*Nitrosomonas*
	$NH_2OH \rightarrow NO_2^-$	*hao*	hydroxylamine dehydrogenase	EC 1.7.2.6	*Nitrosomonas*
	$NO_2^- \rightarrow NO_3^-$	*nxrA*	nitrate reductase/nitrite oxidoreductase, beta subunit	EC 1.7.5.1 1.7.99.4	*Nitrospira, Pseudomonas, Marinobacter*
		nxrB	nitrate reductase/nitrite oxidoreductase, beta subunit	EC 1.7.5.1 1.7.99.4	*Nitrospira, Nitrobacter, Pseudomonas*
Denitrification	$NO_3^- \rightarrow NO_2^-$	*narG, narZ*	nitrate reductase/nitrite oxidoreductase, alpha subunit	EC 1.7.5.1 1.7.99.4	*Microbacterium, Lutibaculum, Tranquillimonas, Emcibacter*
		narH, narY	nitrate reductase/nitrite oxidoreductase, beta subunit	EC 1.7.5.1 1.7.99.4	*Amaricoccus, Microbacterium, Nitratireductor, Nitrococcus*
		narI, narV	nitrate reductase gamma subunit	EC 1.7.5.1 1.7.99.4	*Pseudomonas, Marinobacter, Rhodococcus*
		narJ, narW	nitrate reductase delta subunit	/	*Paracoccus, Agromyces, Litorimicrobium, Ruegeria*
		napB	nitrate reductase (cytochrome), electron transfer subunit	/	*Leisingera, Ruegeria, Yangia*
		napA	periplasmic nitrate reductase	EC 1.7.99.4	*Haloferula, Fuerstia, Bradymonas, Ruegeria*
	$NO_2^- \rightarrow NO$	*nirK*	nitrite reductase (NO-forming)	EC 1.7.2.1	*Ruegeria, Denitromonas, Nitrosomonas, Mesorhizobium*

		nirS	nitrite reductase (NO-forming)/hydroxylamine reductase	EC 1.7.2.1 1.7.99.1	*Pseudomonas, Marinobacter, Ruegeria*
	NO → N_2O	*norB*	nitric oxide reductase subunit B	EC 1.7.2.5	*Pseudomonas*
		norC	nitric oxide reductase subunit C	/	*Ruegeria, Pseudomonas, Nitrosomonas, Marinobacter*
	N_2O → N_2	*nosZ*	nitrous-oxide reductase	EC 1.7.2.4	*Pseudomonas, Marinobacter*
DNRA	NO_3^- → NO_2^-	*narG, narZ*	nitrate reductase/nitrite oxidoreductase, alpha subunit	EC 1.7.5.1 1.7.99.4	*Microbacterium, Lutibaculum, Tranquillimonas, Emcibacter*
		narH, narY	nitrate reductase/nitrite oxidoreductase, beta subunit	EC 1.7.5.1 1.7.99.4	*Amaricoccus, Microbacterium, Nitratireductor, Nitrococcus*
		narI, narV	nitrate reductase gamma subunit	EC 1.7.5.1 1.7.99.4	*Pseudomonas, Marinobacter, Rhodococcus*
		narJ, narW	nitrate reductase delta subunit	/	*Paracoccus, Agromyces, Litorimicrobium, Ruegeria*
		napA	periplasmic nitrate reductase NapA	EC 1.9.6.1	*Ruegeria, Photobacterium, Pseudomonas*
		napB	nitrate reductase (cytochrome), electron transfer subunit	/	*Ruegeria, Photobacterium, Pseudomonas*
	NO_2^- → NH_4^+	*nrfA*	nitrite reductase	EC 1.7.2.2	*Bdellovibrio*
		nrfH	cytochrome c nitrite reductase small subunit	/	*Bdellovibrio*
		nirB	nitrite reductase (NADH) large subunit	EC 1.7.1.15	*Pseudomonas, Ruegeria, Marinobacter, Photobacterium*
		nirD	nitrite reductase (NADH) small subunit	EC 1.7.1.15	*Pseudomonas, Rhodococcus, Marinobacter*
ANRA	NO_3^- → NO_2^-	*nasC, nasA*	assimilatory nitrate reductase catalytic subunit	EC 1.7.99.4	*Pseudomonas, Bacillus, Marinobacter*
		nasB	assimilatory nitrate reductase electron transfer subunit	EC:1.7.99.-	/
		narB	ferredoxin-nitrate reductase	EC 1.7.7.2	*Pseudomonas, Rhodococcus, Marinobacter*
		NR	nitrate reductase (NAD(P)H)	EC:1.7.1.1 1.7.1.2 1.7.1.3	/
	NO_2^- → NH_4^+	*nirA*	ferredoxin-nitrite reductase	EC 1.7.7.1	*Ruegeria, Pseudomonas, Vibrio, Rhodococcus*
		NIT-6	nitrite reductase (NAD(P)H)	EC:1.7.1.4	/
Nitrogen fixation	N_2 → NH_4^+	*nifD*	nitrogenase molybdenum-iron protein alpha chain	EC 1.18.6.1	*Bradyrhizobium*
		nifH	nitrogenase iron protein NifH	EC 1.18.6.1	
		nifK	nitrogenase molybdenum-iron protein beta chain	EC 1.18.6.1	

Ammonium assimilation	$NH_4^+ \rightarrow$ Organic-N	*GLT1*	glutamate synthase (NADPH/NADH)	EC 1.4.1.13 1.4.1.14	/
		gltB	glutamate synthase (NADPH/NADH) large chain	EC 1.4.1.13 1.4.1.14	*Ilumatobacter, Microbacterium, Mycobacterium, Rhodococcus*
		gltD	glutamate synthase (NADPH/NADH) small chain	EC 1.4.1.13 1.4.1.14	*Ruegeria, Vibrio, Hyphomicrobium, Mesorhizobium*
		GLU, gltS	glutamate synthase (ferredoxin)	EC 1.4.7.1	*Photobacterium. Pseudomonas, Hyphomicrobium, Mycobacterium*
		glnA, GLUL	glutamine synthetase	EC 6.3.1.2	*Ruegeria, Pseudomonas, Hyphomicrobium, Methyloceanibacter*
Ammonium production	Organic-N $\rightarrow NH_4^+$	*gudB, rocG*	glutamate dehydrogenase	EC 1.4.1.2	*Bacillus, Microcystis, Rhodopirellula*
		GDH2	glutamate dehydrogenase	EC 1.4.1.2	*Photobacterium, Marinobacter, Pseudomonas, Lysobacter*
		GLUD1_2, gdhA	glutamate dehydrogenase (NAD(P)+)	EC 1.4.1.3	*Ruegeria, Rhodococcus, Microbacterium, Demequina*
		E1.4.1.4, gdhA	glutamate dehydrogenase (NADP+)	EC 1.4.1.4	*Pseudomonas, Microbacterium, Rhodococcus, Demequina*
